# An Integrated Physical, Genetic and Cytogenetic Map of *Brachypodium distachyon*, a Model System for Grass Research

**DOI:** 10.1371/journal.pone.0013461

**Published:** 2010-10-18

**Authors:** Melanie Febrer, Jose Luis Goicoechea, Jonathan Wright, Neil McKenzie, Xiang Song, Jinke Lin, Kristi Collura, Marina Wissotski, Yeisoo Yu, Jetty S. S. Ammiraju, Elzbieta Wolny, Dominika Idziak, Alexander Betekhtin, Dave Kudrna, Robert Hasterok, Rod A. Wing, Michael W. Bevan

**Affiliations:** 1 John Innes Centre, Colney, Norwich, United Kingdom; 2 The Arizona Genomics Institute, School of Plant Sciences and the BIO5 Institute for Collaborative Research, The University of Arizona, Tucson, Arizona, United States of America; 3 Department of Plant Anatomy and Cytology, University of Silesia, Katowice, Poland; University of Toronto, Canada

## Abstract

The pooid subfamily of grasses includes some of the most important crop, forage and turf species, such as wheat, barley and *Lolium*. Developing genomic resources, such as whole-genome physical maps, for analysing the large and complex genomes of these crops and for facilitating biological research in grasses is an important goal in plant biology. We describe a bacterial artificial chromosome (BAC)-based physical map of the wild pooid grass *Brachypodium distachyon* and integrate this with whole genome shotgun sequence (WGS) assemblies using BAC end sequences (BES). The resulting physical map contains 26 contigs spanning the 272 Mb genome. BES from the physical map were also used to integrate a genetic map. This provides an independent vaildation and confirmation of the published WGS assembly. Mapped BACs were used in Fluorescence *In Situ* Hybridisation (FISH) experiments to align the integrated physical map and sequence assemblies to chromosomes with high resolution. The physical, genetic and cytogenetic maps, integrated with whole genome shotgun sequence assemblies, enhance the accuracy and durability of this important genome sequence and will directly facilitate gene isolation.

## Introduction

The diverse and ecologically dominant grass family provides most human and domestic animal nutrition. Members of the Ehrhartoideae (rice), Panicoideae (maize, sorghum) and Pooideae (wheat, barley) grass subfamilies are the main grain crops worldwide and have been extensively improved by selective breeding to enhance productivity and end user qualities. Past yield increases have been achieved by the application of nitrogenous fertilisers, and it is widely accepted that the energy and environmental costs of this and current agronomic practices are no longer sustainable. Furthermore current crops are poorly adapted to possible future variations in climatic conditions. Consequently major efforts to develop new generations of grass crops with higher potential yields from reduced inputs, particularly nitrogenous fertilizers and water, are underway.

Strategies for creating new crops include the use of genomic resources for marker-assisted breeding [1] to incorporate a far wider range of genetic variation into elite breeding lines, and genetic engineering to create crops with enhanced agronomic properties. The complete genome sequence of crop plants is an essential foundation for efficient breeding and gene discovery. Currently the rice [2], maize [3] and sorghum [4] genomes have been sequenced and annotated, and are extensively used for grass crop improvement. However the genomes of pooid grass crops have not yet been sequenced due to their size and complexity, therefore the future improvement of this key group of temperate range crop plants is currently limited by the absence of useful genome resources.

Comparison of gene order in wheat and *Brachypodium sylvaticum*, a wild pooid grass, has shown a high degree of conserved gene order and sequence similarity between orthologous regions of the wheat and *Brachypodium* genomes [5], [6], and genome sequences of *Brachypodium* have been used for the rapid and accurate assembly of a large contig of sequenced wheat BACs [6]. The potential of comparative and functional genomics in temperate grasses using *Brachypodium* sequence has led to the development of genomic resources in the diploid *Brachypodium distachyon*, culminating in the recent production and analysis of a whole genome shotgun sequence assembly [7]. The close similarity of gene sequences between pooid grasses, and the conservation of gene order in diverse grasses, together with the increasing use of *Brachypodium distachyon* (hereafter referred to as *Brachypodium*) as an experimental system, suggest the *Brachypodium* whole genome shotgun sequence assemblies will become an important reference for grass biology. The availability of integrated genome databases, Affymetrix arrays and mapping populations, coupled to its ease and economy of cultivation have all contributed to the development of Brachypodium as an experimental system for temperate grass biology.

Genome-wide physical maps provide a key foundation for gene isolation and for establishing accurate chromosome-scale sequence assemblies. Recent physical mapping approaches use Bacterial Artificial Chromosome (BAC) vectors [8] and High-Information Content Fingerprinting (HICF) methods [9], [10], [11] to generate highly accurate maps rapidly. A BAC-based physical map of *Brachypodium* has been constructed [12], [13] containing 671 contigs covering 410 Mb. This size, compared to the 272 Mb genome size of Brachypodium [7], suggested that many contigs overlapped. BAC end sequences (BES) from this map BAC contigs to the rice genome sequence and identified high collinearity between rice and Brachypodium. Comparison of bin-mapped wheat ESTs [14] with mapped Brachypodium BES identified extensive alignment of some large contigs with wheat chromosomes.

The whole genome shotgun sequence of *Brachypodium* was assembled from end-sequenced clones of different sizes, including BACs from a physical map [12], [13] and from this study. However, physical mapping data was not incorporated into the final assembly. Because physical map data provides invaluable independent assessment of sequence assemblies, both within BACs and for longer-range ordering of contigs, we generated an independent Brachypodium physical map and integrated it with genome sequence assemblies. We also integrated this new physical map with a genetic map [15] and the karyotype to assess and validate the accuracy, coverage and chromosomal location of the whole genome shotgun sequence assemblies [7]. Our analyses independently confirm the whole genome shotgun assemblies and align the linkage map to the physical map and sequence assemblies. We also demonstrate essentially complete coverage of chromosome arms by the physical map and sequence assemblies, and provide an important resource for gene isolation.

## Results

### BAC library construction, fingerprinting, and end-sequencing

Two large-insert BAC libraries of the *Brachypodium distachyon* community standard single- seed descent line Bd21 [16] were constructed from high-molecular weight nuclear DNA. The DNA was partially digested with *Hin*dIII or *Eco*RI, double size selected and ligated into pAGIBAC1 and pIndigoBAC536 *Swa*I, respectively. The *Hin*dIII library (BD_ABa) consisted of 36,864 clones with an average insert size of 128 Kb (Table 1), and the *Eco*RI library (BD_CBa) consisted of 36,864 clones with an average insert size of 124 Kb (Table 1). Together the libraries represent 9.7× genome coverage based on a genome size of 272 Mb [7], [17].

**Table 1:** Characteristics of the two BAC libraries used to construct the *Brachypodium* physical map.

Libraries	Cloning site	Average insert size	No of BACs	No of clones used in mapping	No BAC-end sequences	Genome coverage
BD_ABa	*Hin*dIII	128 Kb	36,864	15,565	34,001	4.5X
BD_CBa	*Eco*RI	124 Kb	36,864	14,947	24,893	4.5X
Total		126 Kb	73,728	30,512	58,894	9.7X

Half of the clones from each library (a total of 36,864 clones) were fingerprinted using the HICF fingerprinting method [11]. A total of 30,512 (82.7%) clones yielded fingerprints suitable for contig assembly (Table 1, Figure S1). Each BAC clone from both libraries was end sequenced, yielding 58,894 BAC-end sequences (BES) and a total of 41 Mb of genome sequence (Table 1).

### Initial build of the physical map

The *Brachypodium* BAC fingerprint data was subjected to overlap analysis using the FingerPrinted Contig software package FPC version 8.9. (See http://www.agcol.arizona.edu/software/fpc/). The initial build of the *Brachypodium* physical map was performed incrementally using a cut-off 1e-50 and a tolerance of 4. The “DQer” function was used to break up all contigs that contained more than 15% of Questionable (Q) clones. The remaining contigs were end-merged by “End to End” function and then singletons were added to the end of contigs by “Singles to End” function (cutoff of 1e-21). The resulting first contig build contained 208 contigs assembled with 30,195 (99%) clones, with 317 (1%) clones remained as singletons (Table 2).

**Table 2:** Main features of the *Brachypodium* physical map.

	Phase I physical map		Phase II physical map
	Automatic contig assembly	After manual editing^1^	After manual editing^2^
Number of clones fingerprinted	30,512	30,512	30,512
Number of clones used for map assembly	30,195	26,800	26,800
Number of singletons	317	472	475
Number of contigs	208	35	26
Contigs containing			
>1000 clones	1	9	10
999–800 clones	1	6	7
799–600 clones	5	6	3
599–400 clones	9	2	2
399–200clones	24	6	1
199–100 clones	45	4	3
<99 clones	123	2	1
Unique bands of the contigs	270,216	253,114	252,810
Physical length of the contigs (Mbp)	324,259	303,736	303,372

1 based on SyMAP alignments with the *Brachypodium* 4X draft sequence

2 based on SyMAP alignments with the *Brachypodium* v1.0 sequence

Contigs were then merged manually by identifying fingerprint overlaps with a lower stringency (1e-15 – 1e-18). Two contigs were merged only if two BAC clones at the end of contigs matched each other in a unique and reciprocal manner, and if these merges were confirmed by alignment of BES with genome sequence assemblies using SyMAP [18]. The 58,894 end sequenced BACs were then aligned with a preliminary assembly of the shotgun sequence of the *Brachypodium* genome (http://www.brachypodium.org/), comprising 1015 super-contigs (SC) representing 260 Mb without gaps, of which 17 were longer than 1 Mb and represented 99% of the genome sequence. This integrated alignment was used to assess and validate the sequence assemblies and to build the physical map further. Two contigs were merged if end-to-end analysis indicated that at least two terminal clones from one contig match at least two terminal clones from another contig. In cases of single pair matches, the alignment of the contigs with the genome sequence helped strengthen the merges and improved the quality of the physical map. In addition, contigs merged in the automatic assembly that did not correspond with the draft sequence were broken and reassembled. As the FPC software creates contigs in a random manner, without taking the order of the contigs into account, contigs were able to be oriented according to BES alignment with the draft genome sequence. After manual editing, the physical map comprised 35 contigs assembled from 26,800 (87.8%) clones, with 472 (1.5%) fingerprints remaining as singletons (Table 2).

In this FPC build, two contigs, named 90 and 1000, had unusual features. Contig 90 contains 537 clones and is highly stacked with 356 buried clones indicating a high level of overlap among the clones. This pattern is typical of BAC clones containing the chloroplast genome [19]. This was confirmed by BESs from these clones showing high BLAST-based sequence similarity to chloroplast genome sequences from *Brachypodium*, rice, wheat and barley (data not shown). Contig 1000 contains 3546 clones spread over only 453 CB units, which is representative of a stack of clones of repeats. This contig was marked as ‘dead contig’ in FPC to avoid possible confusion in future editions of the physical map.

**Figure 1:**
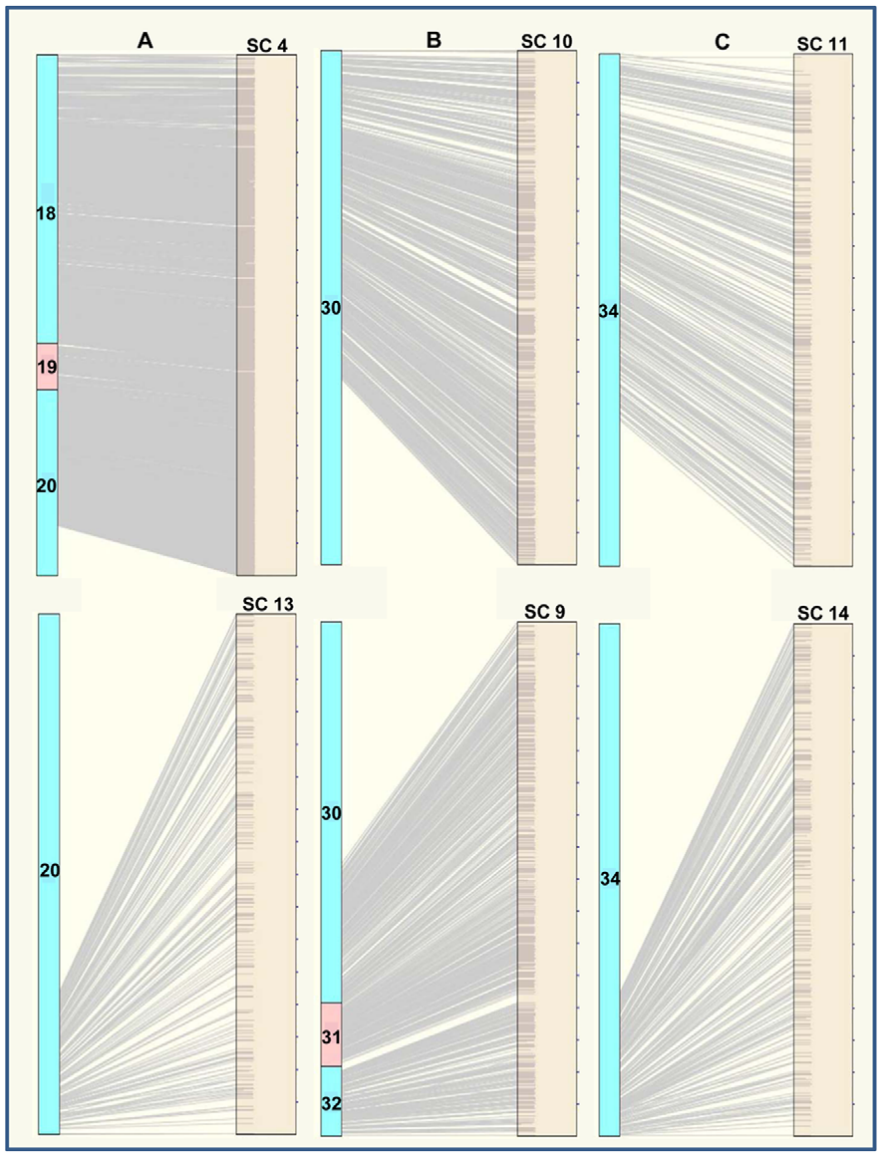
Merging sequence supercontigs (SC) by alignment of the BAC end sequences in the physical map contigs to *Brachypodium* draft genome sequence assemblies. BAC contigs are shown as blue and pink boxes to the left of each alignment, and sequence contigs are shown as beige boxes. The gray lines shown alignments of BAC End Sequences from the BAC contigs to the sequence contigs made using SyMap. Panel A shows that the top of BAC contig 20 aligned with the end of SC4 while the end of BAC contig 20 matched the top of SC13, merging SC4 and SC13. Panel B shows that BAC contig 30 matched SC10 and the top of SC9, merging SC10 and SC9. Panel C shows the top of BAC contig 34 aligned with the end of SC11 while the end of contig 34 matched the top of SC14, merging SC11 and SC14. These 6 SC were merged according to the FPC contig indicated, thus reducing the number of SC in the sequence draft from 17 to 14.

Alignment of the initial physical map with the draft sequence assemblies identified BAC contigs that potentially linked sequence SC (Figure 1). For example, BES from contig 20 of the initial physical map had strong sequence alignments with SC4 and SC13, representing 26.7 Mb and 4.13 Mb of the genome sequence respectively. All the clones of the top part of contig 20 (Figure 1A) matched the end of SC4 and all the clones of the bottom part of contig 20 matched the top of SC13. Based on these BES alignments and the build of contig 20, SC4 and SC13 were merged together forming a new SC of over 30 Mb, thus extending the draft sequence assembly. This was also carried out in two other cases, where SC10 was merged into SC9 giving rise to a new 17 Mb SC (Figure 1B) and SC11 was merged with SC14 resulting in a new 10 Mb SC (Figure 1C). We therefore merged six SC in the preliminary sequence assemblies to create three new assemblies, reducing the number of SC in the draft assembly from 17 to 14, leading to a more extensive and accurate sequence assembly.

### Final physical map

**Figure 2:**
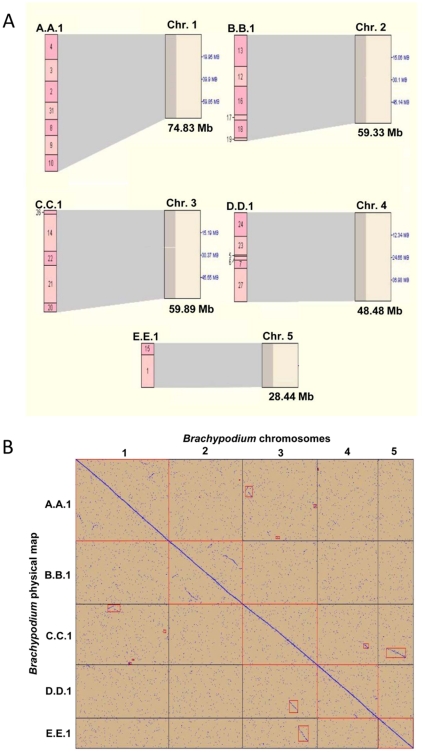
Alignment between the *Brachypodium* physical map and *Brachypodium* v1.0 genome sequence [**7**]. This figure represents a two-way display of the alignment using SyMap. Panel A shows the alignment of fpc- derived physical map contigs (A.A.1 – E.E.1) with the five sequence assemblies (Chr.1-5). Panel B shows a dotplot representation of the alignment between the *Brachypodium* physical map (X axis) and the *Brachypodium* v1.0 genome assemblies (Y axis). The dotplot shows an almost perfect alignment and also shows some segmental duplications and inversions in the *Brachypodium* genome outlined in the small red boxes.

An assembly of the whole genome shotgun sequence of *Brachypodium* has recently been completed and annotated [7]. This sequence was assembled using paired end-read information from the BAC end sequences generated in this study and from Huo *et al* [12], but it did not incorporate any FPC- derived physical mapping data. To assess the published sequence assemblies we aligned them with the FPC-derived physical map using BES. The alignment merged the physical map into 26 contigs with 26,800 (87.8%) clones, with 475 (1.55%) singletons (Table 2, Figure 2A). The final size of the physical map of BACs was 303 Mb based on an approximation of the band size of fingerprints, and BES alignment mapped these contigs onto 271 Mb of the 272 Mb genome. This is consistent with a tendency for band sizes to be over-estimated in fingerprints. The FPC-based physical map of BAC clones independently verified all of the sequence assemblies and long-range scaffolds in the final sequence assembly [7]. This is shown by the complete alignment on the dot plot (Figure 2B) where the 5 large assemblies of the *Brachypodium* v1.0 sequence (Chromosomes 1–5) aligned with the five assemblies of FPC contigs (A.A.1-E.E.1). The dot plot also identified several segmental duplications and inversions (Figure 2B).

### Integration of the BAC-based physical map with a *Brachypodium* genetic map

**Figure 3:**
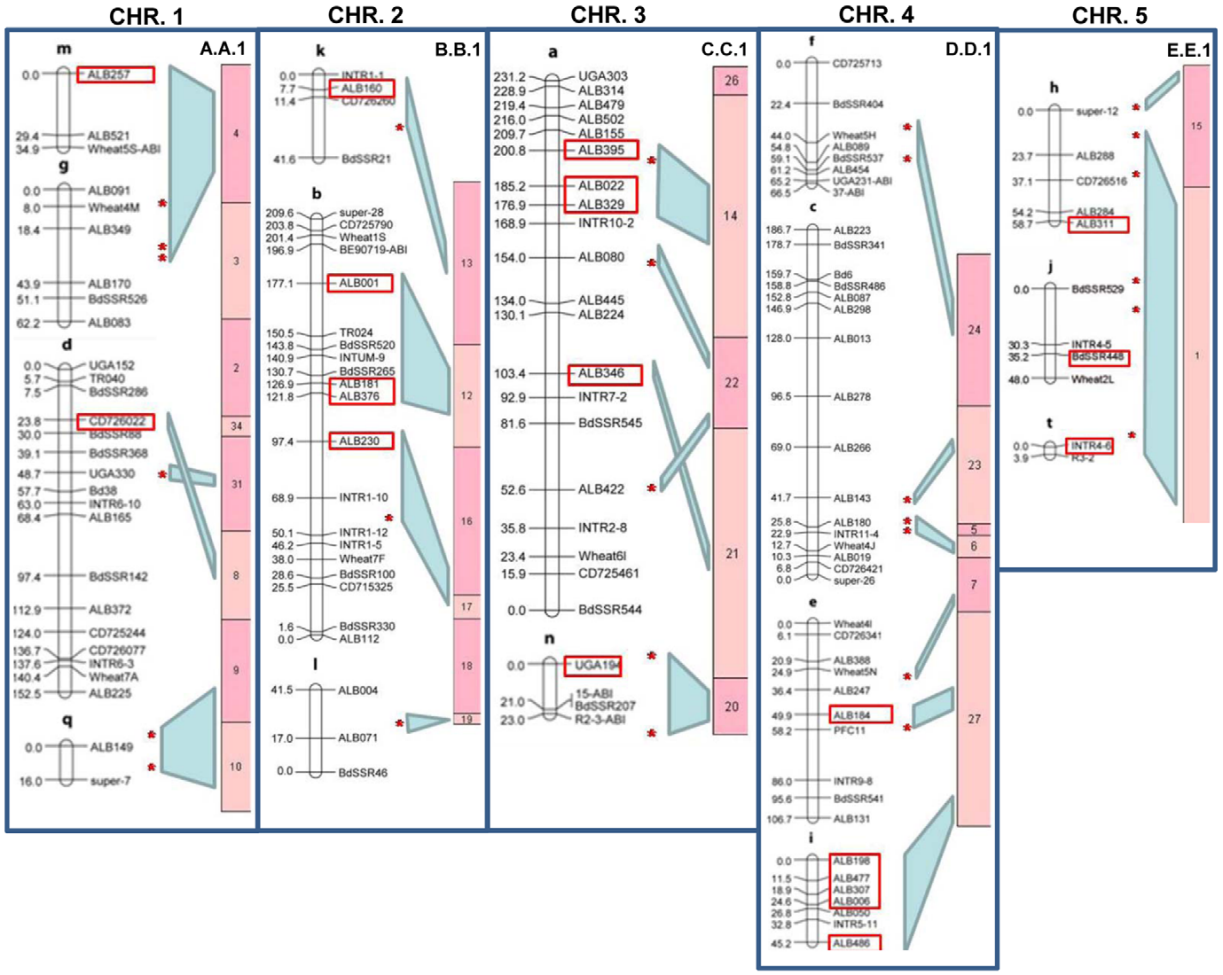
Integration of the genetic and physical maps of *Brachypodium*. The five panels represent each chromosome. Linkage groups [16] are shown on the left of each panel, and the physical maps are shown to the right. The blue panels represent the alignments of the physical and genetic maps according to genetic markers derived from BAC End Sequence (BES) (red asterisks) and from genetically mapped markers mapped to BES by sequence identity (red boxes). The drawings are not to scale.

A genetic map of *Brachypodium distachyon* has been created from 183 F2 individuals derived from a cross between two inbred lines Bd3-1 and Bd21 [15].The map consists of 139 marker loci distributed across 20 linkage groups with a combined length of 1386 cM. To integrate this genetic map with the BAC-based physical map, the sequences of genetic markers were aligned with BES from the physical map using BLAST. This aligned 21 of the genetic markers to the physical map. The alignments were deepened by developing SSR markers from BES of BAC clones located at each end and the middle of each contig in the physical map (Table S1). These markers were mapped onto the same mapping population, and an additional 27 alignments between the genetic and physical maps were identified. Figure 3 shows that 16 of the 20 linkage groups could be aligned with the physical map.

### Chromosomal alignment of the physical map

**Figure 4:**
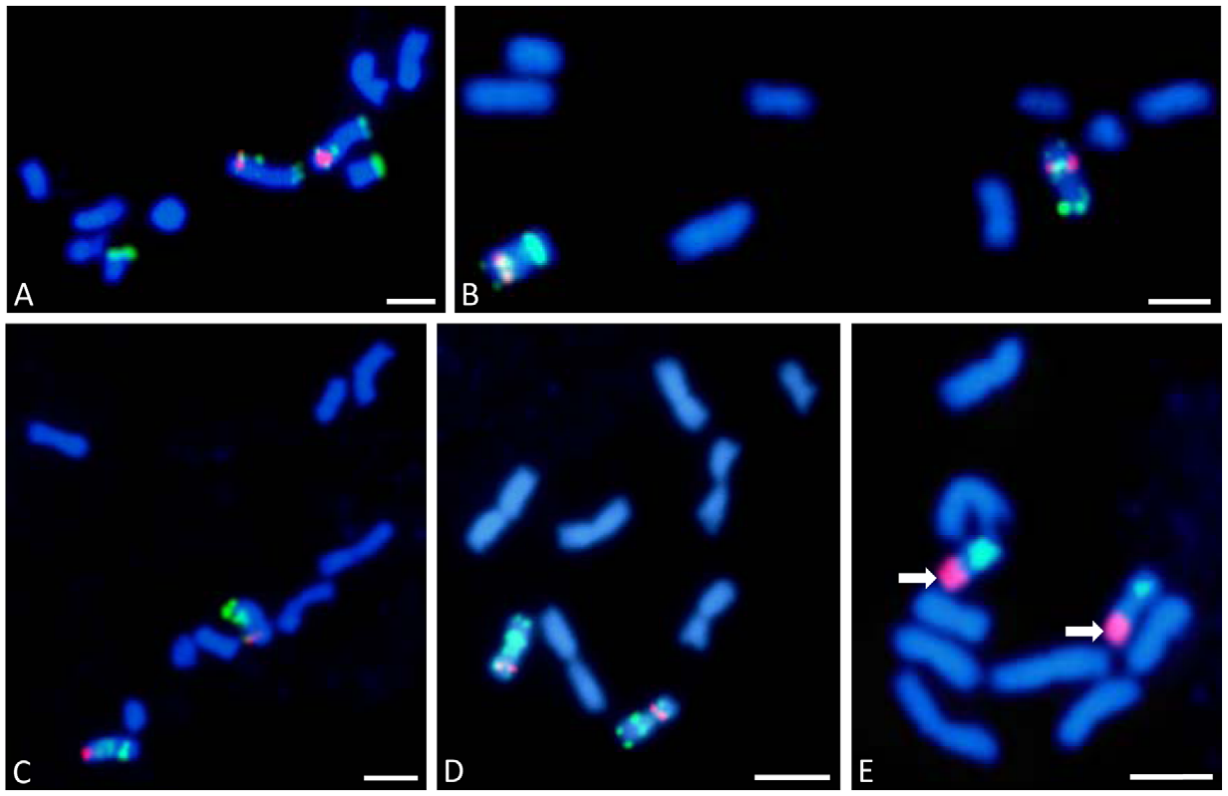
FISH painting of BAC-containing markers of the 5 chromosomes of *Brachypodium* (Bd). **A**. BAC pool of Bd1 (green) with ABR1-26-H1 – anchor for 1L (red). **B**. BAC pool of Bd2 (green) with ABR1-41-E10 – anchor for 2S (red). **C**. BAC pool of Bd3 (green) with ABR5-33-F12– anchor for 3S (red). **D**. BAC pool of Bd4 (green) with ABR5-33-F2 – anchor for 4S (red). **E**. BAC pool of Bd5 (green) with 25S rDNA – anchor for 5S (red) indicated by the white arrows. The details of each BAC pool are described in Table S2. All bars: 5 µm.

**Figure 5:**
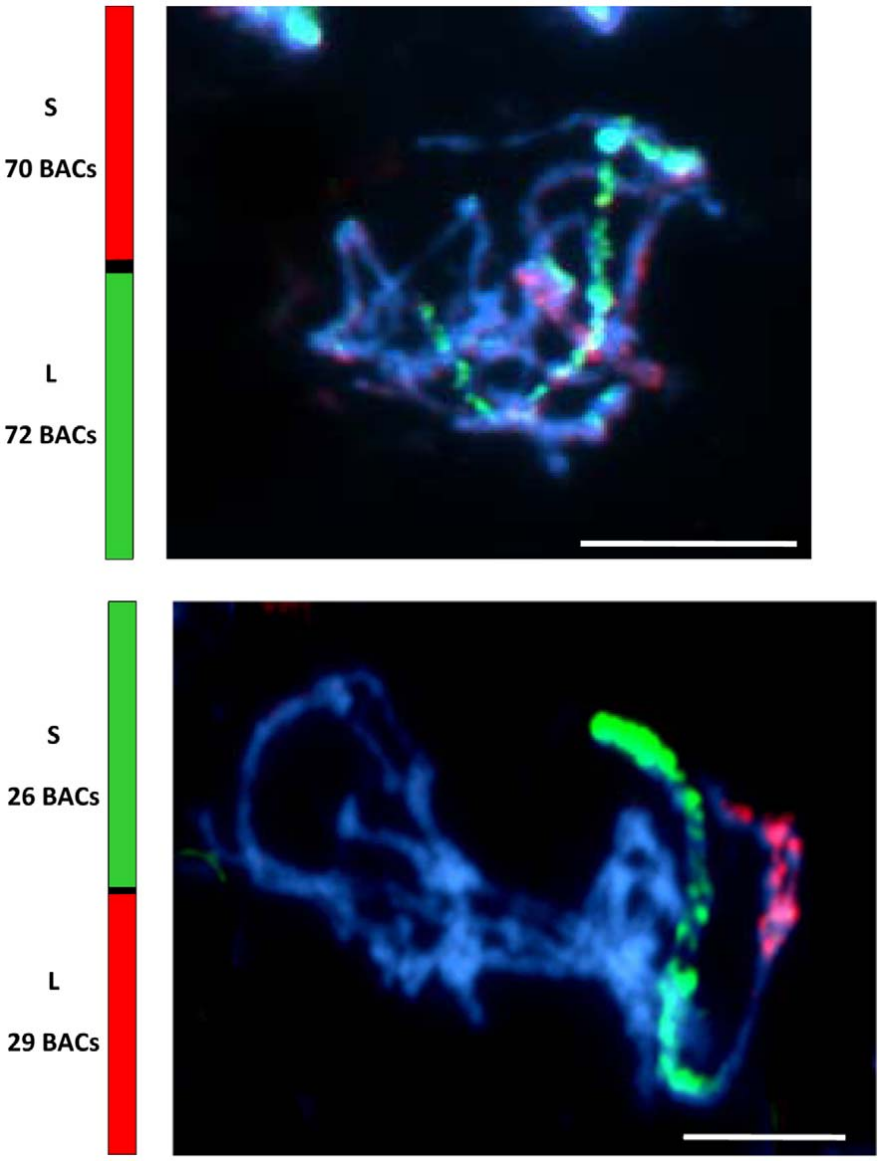
Chromosomal FISH of pachytene chromosome spreads. BACs were selected that spanned physical map assemblies A.A.1 and B.B.1 (see Figure 2). These are described in Table S3. **A**. Alignment with chromosome 1. **B**. Alignment with chromosome 2. Bars: 5 µm.

To define the chromosomal identity of the five assemblies of FPC contigs, BACs from each of the five assemblies (A.A.1-E.E.1) were selected to span a region of each contig assembly and for low repeat content and labelled (Table S2). Each chromosome was identified with a chromosome-specific marker [20]. Figure 4 shows that BAC pools selected from each of the assemblies A.A.1- E.E.1 hybridise specifically to chromosomes 1–5 respectively. The size of the BAC assemblies is also consistent with the size of each chromosome; assembly A.A.1, the largest, corresponded to chromosome 1, and assembly E.E.1, the smallest, corresponded to chromosome 5. To demonstrate the alignment of BAC FPC assemblies with chromosomes, pachytene spreads of Bd 21 were labelled with a pool of BACs (Table S3) representing each predicted arm of chromosomes 1 and 2, the two longest and most easily distinguished chromosomes. In total, 142 BACs were selected for alignment with chromosome 1 and 55 BACs for alignment with chromosome 2. These analyses demonstrated that the order of BACs defined by FPC and by integration with genome sequence assemblies represented entire chromosome arms between 20–38 Mb in length. BAC clones assigned to the pericentromeric regions were omitted in the FISH experiments as their relativelyhigh percentage of repetitive DNA sequences caused extensive cross-hybridisation to non-target chromosomes (data not shown). The absence of these excluded BACs is noticeable on pachytene chromosomes as a blue gap between the red and green signals (Figure 5A and 5B).

## Discussion

The compact and well characterised genome of *Brachypodium distachyon*, the first pooid grass to be sequenced [7], provides an important foundation for analysing and assembling the genome sequences of other pooid grasses, such as wheat and barley, which are characterised by their large size and complexity. The compact growth habit, rapid life cycle, extensive natural variation and abundant seed set have also promoted the development of *Brachypodium* as an experimental system for temperate grass biology. Genetic resources [16], [21] and transformation methods [22], [23] have been developed to exploit the genome sequence to increase our understanding of grass biology for crop improvement.

In this report we describe the construction of a second high quality, high-coverage BAC- based physical map of *Brachypodium* that accounts for 271 Mb of the 272 Mb size genome with 26 large contigs containing nearly 90% of fingerprinted BACs. The *Brachypodium* physical map generated by Gu *et al*, [13] made from a total of 67,151 HinDIII- and BamH1- derived clones (of 100 kb average size), assembled into 671 contigs containing 50,182 BAC clones representing 410 Mb of genome size. In this study, a total of 36,864 HinDIII and EcoR1 clones (average size 128 kb and 124 kb respectively) formed an initial build of 208 contigs containing 30,195 BAC clones representing 303 Mb before alignment with genome sequence contigs. Both assemblies used similar Sulston scores. The significant differences in assembly could be explained by the use of BAC clones that are on average approximately 25% longer in this study, by the use of EcoR1 partial digestion for BAC library production that may clone regions of the genome that are more representative and more easily assembled, and by the use of more stringent criteria for the “end-to-end” function in this study. The Gu *et al* physical map contained many contigs that could potentially be joined, suggesting that the use of longer clones, half of which represent different genome samples, may have contributed to the greater contiguity reported in this study.

Alignment of this physical map with a genetic map of *Brachypodium* [15] identified long-range collinearity between the physical and genetic maps, thereby supporting the physical map assemblies and providing a useful resource for map-based gene isolation by aligning BACs with genetic markers and recombination data. Our physical map has been integrated into a genome browser format and can be accessed through http://www.modelcrop.org. The published *Brachypodium* whole genome shotgun genome sequence [7] was derived by assembly of end- sequence derived from sized multiple insert clones, including many clones from the BAC libraries described in this study. However it did not incorporate physical mapping data to assess contiguity, collinearity and coverage. In this study we describe the integration of an FPC- based physical map with the sequence assemblies using BES. Our analysis confirmed the published assembly by creating a set of 5 independently- derived physical map assemblies. Using fluorescently labelled BACs selected from this physical map in chromosomal FISH experiments, we allocated these physical map assemblies to specific chromosomes on metaphase spreads. The size of the physical map assemblies was consistent with the relative sizes of each chromosome [17]. Furthermore, we aligned four of the largest BAC assemblies to chromosomes arms at high resolution using pachytene spreads and demonstrated that each contig represented essentially a complete chromosome arm. Together, the physical map and our analyses independently verify the whole genome shotgun sequence assemblies and provide a high degree of assurance to the published whole genome shotgun sequence project. The *Brachypodium* genome sequence assemblies therefore provide a reliable and accurate template for understanding the genomes of pooid grass crops such as the cereals wheat and barley, and forage grasses such as *Lolium*. Genome analysis of these temperate range food and feed crops is a high priority for sustainable food production.

## Materials and Methods

### BAC Library Construction and Analysis

All methods were performed essentially as previously described [24], [25]. Young leaf material from 3-week-old *Brachypodium* Bd-21 plants derived by single-seed descent for 9 generations was flash frozen in liquid N_2_ and stored at −80°C for nuclei isolation. Nuclei were purified by density gradient centrifugation and embedded in agarose to form plugs, followed by plug washing, nuclear membrane lysis and storage of High Molecular Weight (HMW) DNA plugs. HMW DNA was partially digested with *Hin*dIII and *Eco*RI, size selected on a 1% CHEF gel, electroeluted, and ligated with dephosphorylated *Hin*dIII (or *Eco*RI) pAGIBAC1 (pIndigoBAC536 *Swa*I). ElectroMax DH10B T1 phage resistant *E. coli* cells were transformed with ligation mix using electroporation, plated on selective LB agar media containing chloramphenicol, X-gal and isopropyl β-d-1-thiogalactopyranoside and incubated overnight at 37°C. Transformed colonies were robotically picked and transferred to bar-coded 384-well microtiter plates, grown at 37°C overnight, and stored at −80°C. The master library and one copy are stored at the BAC/EST Resource Centre of the Arizona Genomics Institute. The libraries are named: BD_ABa (*Hin*dIII) and BD_CBa (*Eco*RI) and are available for public distribution from the AGI ordering website (http://www.genome.arizona.edu/orders/). The average insert size of each library is 128 kb for the *Hin*DIII library and 124 kb for the *Eco*R1 library, as determined by analysing *Not*I restriction digestion profiles of 384 random clones per library on 1% agarose CHEF gels.

### BAC Fingerprinting (FP) and end sequencing (BES)

Half of the clones from each library (a total of 36,864 clones) were fingerprinted using the HICF method [11], involving digestion with five restriction enzymes combinations (*Eco*RI, *Bam*HI, *Xba*I, *Xho*I, and *Hae*III) followed by SNaPshot reagent labelling with four colours at the 3′ ends of the restriction fragments and sizing on a ABI 3730xl DNA analyser using GS1200Liz size standards. The size of DNA fragments from the capillary fingerprinting chromatograms was collected by GeneMapper v3.7 (ABI, Applied Biosystems). Of these fingerprints, 6,352 (17.2%) were removed from the data set due to no insert clones, failure in fingerprinting, clones having fewer than 25 bands or more than 179 bands in the range of 75–500 bp, or cross contamination. Thus, a total of 30,512 (82.7%) clones were successfully fingerprinted for use in contig assembly (Table 1).

Each BAC clone was also end sequenced [26], resulting in a total of 58,894 BAC-end sequences (34,001 for BD_ABa and 24,893 for BD_CBa) (Table 1). This resulted in 41,058,794 bp of high quality sequence of *Brachypodium* genomic DNA, which can be accessed from GenBank accessions FI617783-FI637782 and FI657783-FI671959 for BD_ABa; FI637783-FI657782 and FI671960-FI677907 for BD_CBa.

### Assembling and editing the physical map

Fingerprints of both libraries were assembled using FPC version 8.9 [27] under stringent parameters (cutoff 1e-50 and tolerance 4). The assembly was further refined by contig end merging that used less stringent cutoff values (1e-21) while requiring two overlapping clone pairs for each merge. Questionable clones were removed with the DQer function in three steps, allowing 15% of such clones in the resulting contigs that were automatically merged at cutoff 1e-21 with 61 bands shared by termini clones in the contigs. Finally, singletons were merged at a cutoff of 1e-21. BAC end sequences (BES) in this physical map were masked with RepeatMasker v 3.2.7 [28], using a custom database which integrates repeats from Repbase [29] and plant repeat databases [30] and RetrOryza [31]. Masked BES were aligned to the supercontigs equal or larger than 1 Mb from the *Brachypodium* sequence draft with BLAT[32]. The physical map, BLAT output files and the largest 17 supercontigs (renamed A to Q) were used to create an alignment display with SyMAP software [18]. This integrated alignment data set was used to edit the physical map. Analysis for manual contig merges was performed under more relaxed conditions (cutoff 1e-15 – 1e-18) requiring the same number of shared bands by terminal clones, as in the initial build. Contigs were merged if end-to-end analysis indicated that at least two terminal clones from one contig match at least two terminal clones from another contig. In cases of single pair matches, a merge was performed if the alignment to a sequence contig indicated an overlap. Contigs that indicated a mis-assembly were corrected with the CB function at cutoff 1e-60. The resulting final physical map was used to assess the final sequence assemblies [7] using the procedures described above.

### Integration of the physical map and a genetic map

The final physical map was integrated with a recently published genetic map developed from 183 F2 individuals derived from a cross between the inbred diploid *Brachypodium* lines Bd3-1 and Bd21 [15]. The map consists of 139 marker loci distributed across 20 linkage groups with a total length of 1386 cM. Markers on the genetic map were BLASTed against both the BAC-end sequences of the physical map and the genome sequence assembly to anchor the genetic map to the physical map. New SSR markers were developed from the BAC-end sequences (Table S1). SSR markers were selected at the beginning, middle and end of each physical contig to ensure an even spread of the markers and an optimised integration of both maps. Each marker was first tested for amplification by PCR in the mapping parents and then tested for polymorphism by direct sequencing of the PCR products. The polymorphic markers were then genetically mapped in a subset of the mapping population containing 140 individuals.

### Somatic chromosome preparations, DNA probes and fluorescence *in situ* hybridisation

The preparation of metaphase chromosome spreads from *Brachypodium* line Bd21 root tips was adopted from [33]. The following DNA probes were used in this study: (i) Pooled DNAs of the clones associated with various markers and originated from the *B. distachyon* Bd21 BAC library (Table S2) were labelled by nick translation with digoxigenin-11-dUTP (Roche). (ii) BAC clones of known chromosomal localisation originated from *B. distachyon* ABR1/ABR5 libraries [20] were applied as references and labelled by nick translation with tetramethyl-rhodamine-5-dUTP (Roche). (iii) To visualise the 45S rDNA locus, which is specific and diagnostic for the short arm of chromosome 5, a 2.3-kb *Cla*I subclone of the 25S rDNA coding region of *A. thaliana* [34] was labelled as described above. Pachytene spreads were prepared essentially according to [33]. BACs predicted to span the arms of chromosomes 1 and 2 were identified for use as FISH probes (Table S3). A Perl script was used to get the coordinates of a BAC that make up the *Brachypodium* physical map from the SyMAP database, extract the genomic sequence from the assembly, then RepeatMasker (http://www.repeatmasker.org/) was used with the Pooideae-specific Repeat Element Database (http://mips.helmholtz-muenchen.de/plant/recat/) from MIPS to estimate the repetitive content of the BACs. Low repeat BACs were selected along each arm of A.A.1 and B.B.1 at intervals of 350 kb and 785 kb on average, respectively. Repeat content varied from 0% to 39.2%.

The FISH procedure used a hybridisation mixture according to [35]. Digoxigenated probes were immunodetected using standard protocol for FITC-conjugated anti-digoxigenin antibodies (Roche). The preparations were mounted and counterstained in Vectashield (Vector Laboratories) containing 2.5 µg/ml of 4′,6-diamidino-2-phenylindole (DAPI; Serva). All images were acquired using a *CoolSnap cf* CCD camera (Photometrics) attached to a Leica DMRB epifluorescence microscope. The images were processed uniformly and superimposed using Photoshop (Adobe) software.

## Supporting Information

Figure S1 Example of the clone order fingerprints of a BAC contig of the Brachypodium physical map. Fingerprints are as shown in FPC for Brachypodium contig 20. Each clone is identified above the fingerprint. The scale reads in base pairs for the fragment size.
(0.14 MB DOCX)

Table S1 List of SSR markers designed for the BAC-end sequences mapped onto the genetic map.
(0.05 MB DOC)

Table S2 Identification of BACs from each predicted chromosome used for FISH. The results of this analysis are shown in Figure 4.
(0.01 MB DOCX)

Table S3 Identification of BACs used for aligning physical map assemblies to chromosome arms using FISH. The results of this analysis are shown in Figure 5.
(0.18 MB DOC)
