# Supplementary material for: An Integrated Physical, Genetic and Cytogenetic Map of Brachypodium distachyon, a Model System for Grass Research
Source: PLoS One. 2010 Oct 18;5(10):e13461. doi: 10.1371/journal.pone.0013461 (PMC2956642; doi:10.1371/journal.pone.0013461)

**Figure S1:** Example of the clone order fingerprints of a BAC contig of the Brachypodium physical map. Fingerprints are as shown in FPC for Brachypodium contig 20. Each clone is identified above the fingerprint. The scale reads in base pairs for the fragment size.


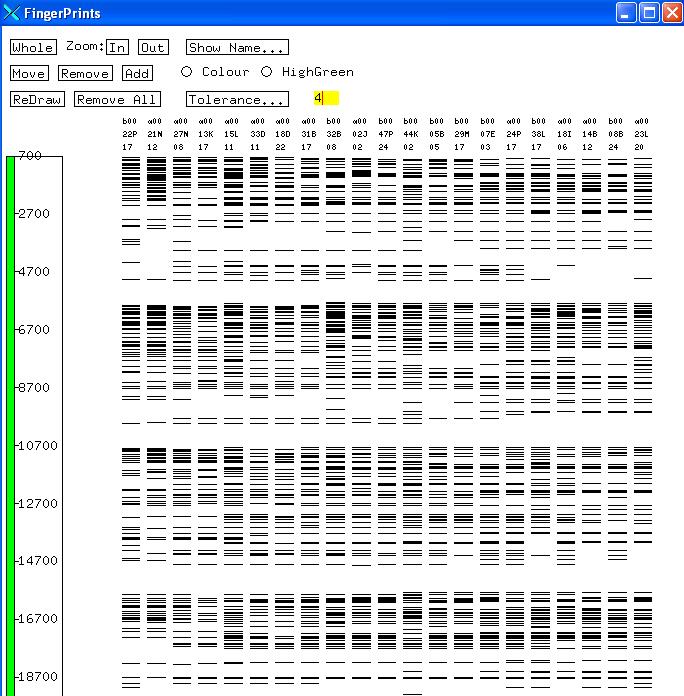

Supplement: Figure S1 — Example of the clone order fingerprints of a BAC contig of the Brachypodium physical map. Fingerprints are as shown in FPC for Brachypodium contig 20. Each clone is identified above the fingerprint. The scale reads in base pairs for the fragment size. (0.14 MB DOCX) [file pone.0013461.s001.docx]
