# Supplementary material for: An Integrated Physical, Genetic and Cytogenetic Map of Brachypodium distachyon, a Model System for Grass Research
Source: PLoS One. 2010 Oct 18;5(10):e13461. doi: 10.1371/journal.pone.0013461 (PMC2956642; doi:10.1371/journal.pone.0013461)
Supplement: Table S1 — List of SSR markers designed for the BAC-end sequences mapped onto the genetic map. (0.05 MB DOC) [file pone.0013461.s002.doc]

**Table S1.**

List of SSR markers designed for the BAC-end sequences mapped onto the genetic map.

| **Marker** | **BAC clone** | **Motif** | **Forward primer** | **Reverse primer** |
| --- | --- | --- | --- | --- |
| BDBES_SSR0108_0001 | a0023D06_r | [GCCG]5 | TGTAAAACGACGGCCAGTAGTCTCCATCGGCTGAATTT | GGTTCCACCAGCAAGTGTCT |
| BDBES_SSR0108_0014 | b0033D02_f | [AAG]9 | TGTAAAACGACGGCCAGTGCATGTACGCACTAGCAAGG | GTTAGCAGGGAGGAGGAAGG |
| BDBES_SSR0108_0034 | a0002N04_f | [TTC]9 | TGTAAAACGACGGCCAGTCAAAGAAACCCCACCAACC | GGACCAGCTTTTACGTGACC |
| BDBES_SSR0108_0223 | a0029E10_f | [CA]27 | TGTAAAACGACGGCCAGTCGTTGCTACCGAGACACCTT | AGCCATTGACGACTTTTTGG |
| BDBES_SSR0108_0323 | b0035H09_r | [AAAG]6 | TGTAAAACGACGGCCAGTACCCAGAAAGATCGGCAGTA | GTGCTTGCATCACGATTCTG |
| BDBES_SSR0108_0332 | a0018K11_r | [TTG]8 | TGTAAAACGACGGCCAGTTCACAAGTGACACCATCCAA | ATGGATCGGAGGAAGTACCA |
| BDBES_SSR0108_0371 | a0036L13_f | [AG]9 | TGTAAAACGACGGCCAGTAGCTAAGCCTGCACCACCTA | GCACACAGGAGAGGATTCGT |
| BDBES_SSR0108_0434 | b0017C21_f | [CA]12 | TGTAAAACGACGGCCAGTTCATGACAAAAACGCGGATA | CTCATGAGTACGGGGAGGAC |
| BDBES_SSR0108_0523 | b0003G22_r | [TCG]5 | TGTAAAACGACGGCCAGTTGAGAAGATGTGTCAGGACAGG | GCTTATGGATTATGGCGATG |
| BDBES_SSR0108_0570 | b0003N09_f | [AC]21 | TGTAAAACGACGGCCAGTGTTTGACCCGGTACCAACAT | AATCCTCGGTGCACGTTTAT |
| BDBES_SSR0108_0712 | a0044B16_r | [CT]13 | TGTAAAACGACGGCCAGTTGGCACAAGAGACGGTGTTA | CCAAGAATCGGGCTAGACAA |
| BDBES_SSR0108_0719 | b0005P05_r | [GA]12 | TGTAAAACGACGGCCAGTGTTGCCCCCTCTGTGTGTAT | CATGGCACATGGTAGGTGAG |
| BDBES_SSR0108_0780 | b0044M23_f | [CCG]8 | TGTAAAACGACGGCCAGTGGCAAAGCCAACAAGGACTA | TAGTTCCGGAGGAGATCACG |
| BDBES_SSR0108_0934 | a0013L16_r | [AT]10 | TGTAAAACGACGGCCAGTCATTCTGATCGGTGCTTGAG | CCGGAGGTACCTAACTGGAG |
| BDBES_SSR0108_1031 | a0019A19_r | [TC]16 | TGTAAAACGACGGCCAGTGCCCCCATATCCCTAACCTA | TCCTGGATTCTCTCCACGAT |
| BDBES_SSR0108_1048 | b0041L12_f | [GCT]7 | TGTAAAACGACGGCCAGTGCTACGATCCGTCACATTCC | TGCCCATTCAACTATCAACAA |
| BDBES_SSR0108_1164 | a0039D05_f | [GAAA]8 | TGTAAAACGACGGCCAGTATTTGGACCGTACGTTTTGC | TGTCGTTCCTCTCGATCCTT |
| BDBES_SSR0108_1169 | a0039O07_f | [TA]8 | TGTAAAACGACGGCCAGTTTTTTGGAATGGTTGTGCAG | CAGCAACTGCGAATGTTTTG |
| BDBES_SSR0108_1189 | a0010O04_f | [GA]14 | TGTAAAACGACGGCCAGTGCTGCAAATAGCACAACGAG | CAAGTCAATTGGGCAAGATG |
| BDBES_SSR0108_1378 | b0047H14_f | [CTG]6 | TGTAAAACGACGGCCAGTGGGGCTAGAGCAGGTCAGTA | TCATGAGCGCATCAGAATTT |
| BDBES_SSR0108_1455 | a0002I07_f | [T]14 | TGTAAAACGACGGCCAGTTGTGATGGAACGACACATCC | CCTCCCGGCTTCATTTTATT |
| BDBES_SSR0108_1673 | a0037L08_f | [T]12 | TGTAAAACGACGGCCAGTTCAGCATGGTAAGCACGAAG | ACCTGCAGCAATCAAATCAG |
| BDBES_SSR0108_1779 | b0041P08_r | [T]15 | TGTAAAACGACGGCCAGTTACCTGCTCTGCTCCATGTG | TCGTTGAACGTCGTCCAGTA |
| BDBES_SSR0108_1786 | b0029E05_r | [T]13 | TGTAAAACGACGGCCAGTGCACGCAACAATTTAAAGCA | AATCTATGCAACGGGCTCAC |
| BDBES_SSR0108_1807 | a0034E14_r | [AT]11 | TGTAAAACGACGGCCAGTGGCAACACACGGACAATGTA | CCCATATTCCCTTGGTCACA |
| BDBES_SSR0108_1819 | b0003P21_f | [GT]11 | TGTAAAACGACGGCCAGTTGTACCCTCATATTCCCCAAA | TCGTTCATTCACTCCACCAA |
| BDBES_SSR0108_1917 | a0003G24_f | [GGC]7 | TGTAAAACGACGGCCAGTCCTTCCCGTCAGAAGACTCA | CAGAGGAGTTCAGGGTGGAG |
| BDBES_SSR0108_1970 | b0040E12_f | [CA]8 | TGTAAAACGACGGCCAGTCGTCAGCTCCTTCCTCTCTC | GGGGAAACGGTTGTGTGTAA |
| BDBES_SSR0108_2004 | b0033O01_f | [TC]9 | TGTAAAACGACGGCCAGTTCTCTGTCTCCCTTCTCTCTGTT | GCCCCACCATGACAGTAAAA |
