# Supplementary material for: An Integrated Physical, Genetic and Cytogenetic Map of Brachypodium distachyon, a Model System for Grass Research
Source: PLoS One. 2010 Oct 18;5(10):e13461. doi: 10.1371/journal.pone.0013461 (PMC2956642; doi:10.1371/journal.pone.0013461)
Supplement: Table S2 — Identification of BACs from each predicted chromosome used for FISH. The results of this analysis are shown in Figure 4. (0.01 MB DOCX) [file pone.0013461.s003.docx]

**Table S2.**

Identification of BACs from each predicted chromosome used for FISH. The results of this analysis are shown in Figure 4.

| **Bd chromosome pool** | **Marker name** | **BAC name** | **Position** |  |  |
| --- | --- | --- | --- | --- | --- |
| 1 | ALB257 | a0040G20 | Bd1:4839832..4990165 | | |
|  | BDBES_SSR0108_0223 | a0029E10 | Bd1:9710460..9852686 | | |
|  | CD726022 | a0030F11 | Bd1:28021549..28174061 | | |
|  | BDBES_SSR0108_0523 | b0003G22 | Bd1:68456284..68584554 | | |
|  |  |  |  |  |  |
| 2 | ALB160 | a0044B16 | Bd2:8154609..8305485 | | |
|  | BDBES_SSR0108_0719 | b0005P05 | Bd2:7745519..7891999 | | |
|  | BDBES_SSR0108_0712 | a0044B16 | Bd2:8154609..8305485 | | |
|  | ALB181 | b0023P23 | Bd2:13336480..13486307 | | |
|  | BDBES_SSR0108_1031 | a0019A19 | Bd2:42120506..42313575 | | |
|  | BDBES_SSR0108_1164 | a0039D05 | Bd2:59108568..59255712 | | |
|  |  |  |  |  |  |
| 3 | ALB346 | a0004A14 | Bd3:39998994..40158254 | | |
|  | BDBES_SSR0108_1189 | a0010O04 | Bd3:56897060..57037750 | | |
|  | UGA194 | a0041J04 | Bd3:57561061..57599810 | | |
|  | BDBES_SSR0108_1169 | a0039O07 | Bd3:59536628..59694761 | | |
|  |  |  |  |  |  |
| 4 | BDBES_SSR0108_2004 | b0033O01 | Bd4:5392842..5554007 | | |
|  | BDBES_SSR0108_1455 | a0002I07 | Bd4:10790233..10939500 | | |
|  | BDBES_SSR0108_0332 | a0018K11 | Bd4:25359516..25517839 | | |
|  | BDBES_SSR0108_0371 | a0036L13 | Bd4:29990960..30166954 | | |
|  | BDBES_SSR0108_1673 | a0037L08 | Bd4:31755113..31940803 | | |
|  | ALB198 | a0045G06 | Bd4:46843082..46994022 | | |
|  | ALB006 | b0016N17 | Bd4:46592449..46762416 | | |
|  | ALB486 | a0022E14 | Bd4:46773621..46913354 | | |
|  |  |  |  |  |  |
| 5 | BDBES_SSR0108_0934 | a0013L16 | Bd5:740720..909223 | |  |
|  | BDBES_SSR0108_0014 | b0033D02 | Bd5:27954962..28064281 | | |
|  | BDBES_SSR0108_0570 | b0003N09 | Bd5:9790075..9874961 | | |
|  | ALB311 | a0015J02 | Bd5:18580275..18736126 | | |
|  | INTR4-6 | b0013P03 | Bd5:27959863..28112239 | | |
|  | BDBES_SSR0108_0034 | a0002N04 | Bd5:26766263..26917780 | | |
|  | BDBES_SSR0108_1786 | b0029E05 | Bd5:19821996..19956957 | | |
|  | BDBES_SSR0108_1779 | b0041P08 | Bd5:18721312..18901201 | | |
|  |  |  |  |  |  |
