# Supplementary material for: An Integrated Physical, Genetic and Cytogenetic Map of Brachypodium distachyon, a Model System for Grass Research
Source: PLoS One. 2010 Oct 18;5(10):e13461. doi: 10.1371/journal.pone.0013461 (PMC2956642; doi:10.1371/journal.pone.0013461)
Supplement: Table S3 — Identification of BACs used for aligning physical map assemblies to chromosome arms using FISH. The results of this analysis are shown in Figure 5. (0.18 MB DOC) [file pone.0013461.s004.doc]

**Table S3.** Identification of BACs used for aligning physical map assemblies to chromosome arms using FISH. The results of this analysis are shown in Figure 5.

| **Clone ID** | **Position** | **Repeats** |
| --- | --- | --- |
| a0035K02 | Bd1S: 147863: 304506 | 18,31% |
| b0027N17 | Bd1S: 560624: 710332 | 6,56% |
| a0037D23 | Bd1S: 1171403: 1328435 | 13,07% |
| a0012F06 | Bd1S: 1537097: 1734409 | 7,59% |
| a0032E05 | Bd1S: 1907231: 2063694 | 11,83% |
| a0008O14 | Bd1S: 2635548: 2801693 | 8,30% |
| a0021B03 | Bd1S: 3028832: 3173186 | 6,23% |
| a0004B12 | Bd1S: 3276891: 3460444 | 5,68% |
| b0044D24 | Bd1S: 3878248: 4004060 | 13,91% |
| b0003A11 | Bd1S: 4404030: 4546882 | 30,82% |
| a0032K13 | Bd1S: 5048843: 5206517 | 26,89% |
| a0017K22 | Bd1S: 5375697: 5509098 | 17,71% |
| b0037O18 | Bd1S: 6122656: 6272292 | 19,17% |
| a0022C04 | Bd1S: 6574012: 6741405 | 30,61% |
| b0040G07 | Bd1S: 7221475: 7389553 | 30,09% |
| b0013P15 | Bd1S: 7656602: 7805960 | 29,21% |
| a0013O16 | Bd1S: 7926543: 8055682 | 39,20% |
| b0030L10 | Bd1S: 8680898: 8845282 | 10,03% |
| b0012L20 | Bd1S: 8850673: 9007358 | 14,08% |
| a0015H06 | Bd1S: 9424591: 9569999 | 14,80% |
| a0007G23 | Bd1S: 9965773: 10101237 | 15,91% |
| a0032D10 | Bd1S: 10490542: 10652198 | 11,18% |
| a0003J21 | Bd1S: 10927667: 11073109 | 16,93% |
| a0023P13 | Bd1S: 11505702: 11632287 | 22,48% |
| a0032F08 | Bd1S: 12115980: 12241228 | 17,12% |
| a0024M10 | Bd1S: 12444261: 12575731 | 23,81% |
| a0027D04 | Bd1S: 12706461: 12847057 | 22,87% |
| b0019G20 | Bd1S: 13362834: 13517753 | 21,02% |
| b0023C02 | Bd1S: 13999817: 14137163 | 13,45% |
| b0001G04 | Bd1S: 14561692: 14709543 | 15,53% |
| a0020A04 | Bd1S: 15092918: 15238493 | 0,00% |
| b0023O18 | Bd1S: 15449374: 15577903 | 0,00% |
| a0003N21 | Bd1S: 16107590: 16251236 | 10,80% |
| a0002N01 | Bd1S: 16344468: 16496573 | 11,12% |
| a0009N18 | Bd1S: 17150298: 17335777 | 0,00% |
| a0014L23 | Bd1S: 17404191: 17543242 | 7,86% |
| b0018P22 | Bd1S: 18190466: 18326563 | 23,15% |
| a0017E13 | Bd1S: 18574112: 18708723 | 0,00% |
| a0010I03 | Bd1S: 19198770: 19342731 | 7,06% |
| a0007L04 | Bd1S: 19364297: 19511432 | 8,67% |
| b0002O16 | Bd1S: 20013520: 20160236 | 0,00% |
| a0024N14 | Bd1S: 20488400: 20631457 | 19,18% |
| a0027K03 | Bd1S: 21168673: 21307307 | 0,00% |
| a0010K04 | Bd1S: 21496092: 21643627 | 19,83% |
| a0011I01 | Bd1S: 21907910: 22040598 | 0,00% |
| a0018B03 | Bd1S: 22412015: 22565632 | 32,71% |
| b0022H13 | Bd1S: 23114454: 23242441 | 0,00% |
| a0023E14 | Bd1S: 23230575: 23392276 | 21,52% |
| a0043B06 | Bd1S: 24028749: 24191469 | 22,76% |
| a0042C21 | Bd1S: 24228323: 24375228 | 16,48% |
| a0026E19 | Bd1S: 25017625: 25161139 | 14,31% |
| a0046B12 | Bd1S: 25556278: 25718695 | 17,60% |
| a0018O15 | Bd1S: 25727688: 25878318 | 13,18% |
| b0028A06 | Bd1S: 26442023: 26591576 | 16,02% |
| b0002C04 | Bd1S: 27060765: 27214938 | 27,84% |
| a0006K13 | Bd1S: 27522409: 27682274 | 23,09% |
| a0044I06 | Bd1S: 28135872: 28292480 | 21,81% |
| a0043P17 | Bd1S: 28526824: 28683718 | 21,98% |
| a0029A09 | Bd1S: 28940084: 29079192 | 22,18% |
| a0032C01 | Bd1S: 29475135: 29677346 | 31,42% |
| a0002G12 | Bd1S: 30075959: 30187436 | 25,08% |
| a0007B20 | Bd1S: 30824142: 30835981 | 8,00% |
| a0036J15 | Bd1S: 31222238: 31387974 | 13,86% |
| a0037D16 | Bd1S: 31313973: 31498115 | 24,11% |
| a0018G20 | Bd1S: 32094917: 32274028 | 12,54% |
| b0024I19 | Bd1S: 32507293: 32633286 | 0,00% |
| b0014O02 | Bd1S: 33010624: 33123772 | 0,00% |
| a0024G16 | Bd1S: 33587038: 33745554 | 0,00% |
| b0037O03 | Bd1S: 33832455: 34023561 | 20,87% |
| a0004L01 | Bd1S: 34316249: 34466638 | 15,53% |
| b0011C11 | Bd1L: 38625084: 38768816 | 25,98% |
| a0002G19 | Bd1L: 39219901: 39352626 | 20,11% |
| a0030K01 | Bd1L: 39352642: 39424325 | 13,60% |
| a0002I22 | Bd1L: 39952805: 40102980 | 19,01% |
| b0023K21 | Bd1L: 40363127: 40508554 | 24,17% |
| a0003G01 | Bd1L: 41070484: 41199669 | 5,68% |
| a0017I18 | Bd1L: 41400831: 41536057 | 10,35% |
| a0006H08 | Bd1L: 42291978: 42445395 | 30,82% |
| a0034B17 | Bd1L: 42516220: 42665540 | 26,89% |
| a0022M24 | Bd1L: 43211660: 43355407 | 17,71% |
| b0004P09 | Bd1L: 43536825: 43670757 | 0,00% |
| b0003O14 | Bd1L: 43968448: 44100412 | 0,00% |
| a0046B04 | Bd1L: 44701450: 44835069 | 28,04% |
| a0009H21 | Bd1L: 45130478: 45275483 | 30,09% |
| b0019N18 | Bd1L: 45624834: 45763826 | 29,21% |
| b0039A23 | Bd1L: 45904934: 46085862 | 34,61% |
| b0025P22 | Bd1L: 46564769: 46692954 | 10,03% |
| a0045K11 | Bd1L: 47017729: 47159607 | 14,08% |
| a0018I01 | Bd1L: 47327837: 47472575 | 14,80% |
| a0018A03 | Bd1L: 48153393: 48351371 | 12,13% |
| b0044L08 | Bd1L: 48612347: 48783561 | 9,01% |
| a0016L07 | Bd1L: 49131764: 49282548 | 17,24% |
| b0042L08 | Bd1L: 49567141: 49756854 | 22,48% |
| a0003G14 | Bd1L: 50139085: 50274374 | 17,12% |
| a0007A17 | Bd1L: 50488742: 50629310 | 23,81% |
| a0002G03 | Bd1L: 50987420: 51131768 | 21,02% |
| a0002M19 | Bd1L: 51404954: 51599184 | 13,45% |
| b0035K24 | Bd1L: 51720482: 51914140 | 15,53% |
| a0046C24 | Bd1L: 52577876: 52717406 | 12,29% |
| a0020C13 | Bd1L: 52998818: 53110321 | 14,83% |
| a0011F10 | Bd1L: 53395079: 53532078 | 10,55% |
| a0046G17 | Bd1L: 54082210: 54253048 | 0,00% |
| b0047M09 | Bd1L: 54775761: 54934862 | 11,12% |
| b0036M23 | Bd1L: 55099293: 55274619 | 14,70% |
| a0011D03 | Bd1L: 55361479: 55401129 | 7,86% |
| b0044C20 | Bd1L: 56154348: 56285508 | 16,71% |
| b0013C18 | Bd1L: 56402015: 56523891 | 0,00% |
| b0028P17 | Bd1L: 57093738: 57225377 | 0,00% |
| a0022N20 | Bd1L: 57208701: 57348499 | 11,96% |
| a0019B04 | Bd1L: 58011832: 58155264 | 19,18% |
| b0003K21 | Bd1L: 58350464: 58480351 | 15,82% |
| a0010A14 | Bd1L: 59503419: 59676696 | 0,00% |
| a0008E12 | Bd1L: 60079495: 60251020 | 17,16% |
| a0005H16 | Bd1L: 60258365: 60413281 | 0,00% |
| b0037A14 | Bd1L: 61095306: 61288697 | 19,17% |
| a0012H18 | Bd1L: 61475648: 61619144 | 0,00% |
| b0003A21 | Bd1L: 61920716: 62077069 | 0,00% |
| a0034M17 | Bd1L: 62501248: 62642447 | 0,00% |
| a0045D19 | Bd1L: 63062019: 63221983 | 14,31% |
| b0022G04 | Bd1L: 63557791: 63711230 | 17,60% |
| a0013D23 | Bd1L: 64120769: 64297730 | 16,02% |
| b0011I02 | Bd1L: 64559074: 64702171 | 27,84% |
| a0003I14 | Bd1L: 65067565: 65202176 | 23,09% |
| a0046P14 | Bd1L: 65376014: 65522455 | 21,81% |
| a0009I15 | Bd1L: 65946210: 66098108 | 21,98% |
| b0026H13 | Bd1L: 66197674: 66346594 | 22,18% |
| b0030D22 | Bd1L: 67065313: 67205367 | 25,08% |
| a0019B19 | Bd1L: 67392232: 67529032 | 8,00% |
| b0003K24 | Bd1L: 67945518: 68072820 | 13,86% |
| a0011O07 | Bd1L: 68533765: 68686250 | 24,11% |
| a0043A05 | Bd1L: 68898017: 69053532 | 12,54% |
| b0004O01 | Bd1L: 69023274: 69164463 | 23,47% |
| b0039M08 | Bd1L: 69966292: 70146601 | 29,34% |
| a0040G14 | Bd1L: 70435911: 70578835 | 17,94% |
| b0017K19 | Bd1L: 71146553: 71281318 | 0,00% |
| a0021F18 | Bd1L: 71455475: 71597258 | 0,00% |
| a0041A08 | Bd1L: 72027767: 72181888 | 6,97% |
| b0002N07 | Bd1L: 72465040: 72619352 | 4,45% |
| a0005K09 | Bd1L: 72948475: 73083942 | 4,87% |
| b0039K17 | Bd1L: 73601518: 73740071 | 5,12% |
| a0033F06 | Bd1L: 74020535: 74180685 | 4,77% |
| b0035K23 | Bd1L: 74475472: 74659792 | 12,79% |
| a0038A01 | Bd2S: 1022: 132144 | 4,73% |
| a0026H23 | Bd2S: 501743: 631176 | 5,15% |
| b0039C09 | Bd2S: 1311448: 1507438 | 6,21% |
| a0027K15 | Bd2S: 1864643: 2004976 | 4,27% |
| b0035C01 | Bd2S: 2500100: 2659222 | 4,28% |
| b0002F19 | Bd2S: 2858161: 3010741 | 5,64% |
| a0028O04 | Bd2S: 3492740: 3587755 | 10,20% |
| b0048M15 | Bd2S: 3999943: 4170302 | 18,58% |
| a0002P22 | Bd2S: 4319740: 4509765 | 7,60% |
| a0045F24 | Bd2S: 6004397: 6146555 | 12,87% |
| a0047M10 | Bd2S: 7007047: 7144308 | 8,94 |
| a0019E04 | Bd2S: 8843922: 9006117 | 13,8 |
| a0012B07 | Bd2S: 9006125: 9148678 | 6,31 |
| a0005E09 | Bd2S: 10380990: 10507985 | 10,31 |
| b0048L18 | Bd2S: 12984227: 13000547 | 8,85 |
| a0044D02 | Bd2S: 14006553: 14195269 | 11,1 |
| a0017D02 | Bd2S: 15866689: 16021967 | 17,9 |
| a0031J04 | Bd2S: 16021976: 16162135 | 12,06 |
| a0047D12 | Bd2S: 17856422: 17996794 | 27,25 |
| a0021H13 | Bd2S: 18176419: 18323680 | 28,06 |
| a0026K14 | Bd2S: 19861012: 20005795 | 15,73 |
| a0043C22 | Bd2S: 20005803: 20143867 | 25,91 |
| b0006D07 | Bd2S: 20880418: 21008785 | 17,52 |
| b0015N23 | Bd2S: 21980992: 22118800 | 12,71 |
| b0038L02 | Bd2S: 22509927: 22639901 | 23,44 |
| b0011O19 | Bd2S: 25393226: 25510444 | 14,12 |
| b0031J17 | Bd2L: 33543920: 33664342 | 15,14% |
| a0014K11 | Bd2L: 34309867: 34503922 | 8,53% |
| b0003D21 | Bd2L: 35507055: 35522066 | 19,01% |
| b0007E06 | Bd2L: 36376507: 36505573 | 18,82% |
| b0022I07 | Bd2L: 38509106: 38646001 | 9,09% |
| a0024L09 | Bd2L: 38997728: 39192842 | 8,39% |
| b0031K20 | Bd2L: 39779799: 39931474 | 5,43% |
| b0016E24 | Bd2L: 39997753: 40003453 | 1,65% |
| a0043N06 | Bd2L: 41508421: 41714064 | 18,78% |
| a0008H07 | Bd2L: 42500887: 42664133 | 14,08% |
| b0018H13 | Bd2L: 42943153: 43001509 | 9,64% |
| a0029H05 | Bd2L: 43505258: 43648404 | 13,83% |
| a0009N24 | Bd2L: 44005924: 44173470 | 13,71% |
| b0041G17 | Bd2L: 44876290: 45007631 | 13,75% |
| b0031I09 | Bd2L: 46500135: 46639653 | 7,51% |
| b0040K17 | Bd2L: 47000159: 47021312 | 4,78% |
| b0019P09 | Bd2L: 48369110: 48504229 | 3,43% |
| b0041J21 | Bd2L: 49505774: 49706051 | 9,11% |
| b0038L04 | Bd2L: 50005019: 50143082 | 5,40% |
| b0012J01 | Bd2L: 51003290: 51006240 | 0,00% |
| a0031O24 | Bd2L: 52001822: 52162247 | 4,16% |
| a0036P06 | Bd2L: 52875522: 53003468 | 5,61% |
| a0027O24 | Bd2L: 53007896: 53168487 | 8,87% |
| b0040O17 | Bd2L: 53370233: 53504052 | 4,56% |
| b0036G07 | Bd2L: 53816466: 54010118 | 3,84% |
| b0027N08 | Bd2L: 54420021: 54540398 | 9,29% |
| b0047O03 | Bd2L: 55698147: 55846468 | 5,48% |
| a0038M22 | Bd2L: 56336703: 56502216 | 4,16% |
| a0038G14 | Bd2L: 57002804: 57148130 | 7,24% |

S – short arm. L – long arm
